# Two New Species of *Sarcophaga* (*Sarcophaga*) Meigen, 1826 (Diptera: Sarcophagidae) from Türkiye, with a Key to the Turkish Species †

**DOI:** 10.3390/insects17060546

**Published:** 2026-05-23

**Authors:** Gamze Pekbey, Thomas Pape

**Affiliations:** 1 Department of Plant Protection, Faculty of Agriculture, Yozgat Bozok University, 66100 Yozgat, Türkiye; 2 Natural History Museum Denmark, Science Faculty, University of Copenhagen, 2100 Copenhagen, Denmark; tpape@snm.ku.dk

**Keywords:** flesh flies, male terminalia, Palaearctic, morphology, biodiversity, Anatolia, SDG 15

## Abstract

Two new species of the flesh fly subgenus *Sarcophaga* Meigen, 1826 (Diptera: Sarcophagidae), *Sarcophaga* (*S*.) *karai* sp. nov. and *Sarcophaga* (*S*.) *hayati* sp. nov., are described from Türkiye. For each new taxon, detailed morphological descriptions, differential diagnoses, and high-resolution illustrations of the male terminalia are provided. The genital structures of the newly recognized taxa were further examined using scanning electron microscopy (SEM), allowing detailed characterization of surface morphology and fine ultrastructural features not discernible with conventional optical methods. The diagnostic value of key morphological structures of the male terminalia is discussed in the context of species delimitation within the subgenus. Each new species is compared with those most similar in terminalia morphology, and an updated identification key to the males of Turkish representatives of the subgenus is provided to facilitate future faunistic studies in the region. These findings contribute to a more comprehensive understanding of sarcophagid diversity across the Anatolian Peninsula and highlight the substantial species richness that remains to be documented within this group.

## Simple Summary

Flesh flies of the subgenus *Sarcophaga* Meigen, 1826 (Diptera: Sarcophagidae) are ecologically important insects found worldwide. Despite their significance, the diversity of this group in Türkiye remains incompletely documented. In this study, two species are described as new: *Sarcophaga* (*S.*) *karai* sp. nov. and *Sarcophaga* (*S.*) *hayati* sp. nov., both collected from Türkiye. The male terminalia were further investigated by scanning electron microscopy. Detailed morphological descriptions, differential diagnoses, and illustrations of the male terminalia are provided, and each new species is compared with the morphologically most similar species. An identification key is provided to help researchers identify all known male representatives of this fly group recorded from Türkiye.

## 1. Introduction

Flesh flies (Diptera: Sarcophagidae) are a diverse and ecologically important group of insects, many of which are involved in decomposition and have applications in forensic science, medicine, and ecology [1,2]. The genus *Sarcophaga* Meigen, 1826 is especially species-rich and taxonomically complex, particularly in the Palaearctic Region. This complexity, combined with the group’s notorious morphological uniformity, has significantly delayed progress in documenting diversity and distribution [3]. Because adults exhibit highly similar external features, reliable species-level identification typically depends on fine structural differences in the male terminalia, leading most faunistic surveys and morphology-based phylogenetic studies to rely primarily, or even exclusively, on male specimens, while females remain poorly characterized or effectively unidentifiable in many taxa [4,5]. This longstanding dependence on the morphology of male terminalia has shaped both traditional taxonomy and modern revisions, motivating detailed redescriptions, male-terminalia-focused identification keys, and integrative approaches combining morphology with molecular tools to refine species boundaries and improve the utility of *Sarcophaga* in applied entomology and biodiversity research [4,6,7].

The nominotypical subgenus *Sarcophaga* Meigen, 1826 currently comprises over 30 described species in the western Palaearctic [5], forming an ecologically and taxonomically important component of the regional sarcophagid fauna. Species delimitation has traditionally relied on detailed examination of male terminalia, especially the structure of the phallic complex, including the distiphallus, vesica, and associated sclerites, which provide consistent interspecific differences and remain the primary basis for taxonomic work in the group. In contrast, females of closely related species are often difficult to distinguish morphologically and are in many cases considered indistinguishable based on external characters alone [1,4,5].

At the subgenus level, *Sarcophaga* (*Sarcophaga*) can be identified primarily by the structure of the male phallic complex, especially the configuration of the distiphallus, vesica, harpes, juxta, and associated lateral styli. Keys and diagnoses provided by Pape [8], Povolný and Verves [9], and Richet et al. [10] remain the principal references for subgenus-level identification.

Species of the subgenus are predominantly associated with earthworms, with larvae acting as predators or parasitoids of Lumbricidae, typically attacking hosts near the clitellum [8,9]. While certain species, including *Sarcophaga* (*S.*) *carnaria* (Linnaeus, 1758), *S*. (*S*.) *subvicina* Rohdendorf, 1937, and *S. (S.) variegata* (Scopoli, 1763) can complete development under laboratory conditions on meat, liver, or other decaying animal tissue, this capacity most likely reflects facultative behavioural flexibility rather than an accurate representation of their natural ecological biology [11]. Adults occupy a wide range of open and semi-open habitats, from forest edges and agricultural landscapes to anthropogenically modified environments, reflecting an ecological gradient from specialized invertebrate-associated species to more euryecious taxa [8,9,11].

Several widespread species, including *Sarcophaga* (*S.*) *carnaria* (Linnaeus, 1758), *S*. (*S*.) *lehmanni* Mueller, 1922, *S*. (*S.*) *subvicina* Rohdendorf, 1937, and *S*. (*S*.) *variegata* (Scopoli, 1763), show extensive overlap in most morphological traits, with only a limited number of characters providing partial diagnostic value [5]. The increasing use of molecular approaches such as DNA barcoding has not completely resolved these problems, as molecular reference libraries are still sparsely populated and studies have shown reduced genetic differentiation among closely related flesh fly species, likely reflecting relatively recent evolutionary diversification and incomplete lineage divergence [6,7,12,13]. Ultimately, a robust systematic framework for the family requires an integrated approach encompassing multiple genetic markers, comprehensive reference libraries, and expert morphological revision [12,13,14].

Given these limitations, morphology-based analyses focusing on structures of male terminalia remain essential for reliable species identification. The use of standardized, discrete characters is particularly important in regional studies where closely related taxa often coexist and require consistent criteria for differentiation. In this context, scanning electron microscopy (SEM) has proven valuable for revealing fine ultrastructural details that are not resolvable under stereomicroscopy, enabling more precise characterization of surface textures, micro-ornamentation, and three-dimensional configurations of complex sclerites [6,7,15].

This study addresses this gap by describing two new species of the subgenus *Sarcophaga* Meigen, 1826 from Türkiye, *S*. (*S*.) *karai* sp. nov. and *S*. (*S*.) *hayati* sp. nov., based on detailed morphological examination of male terminalia supported by scanning electron microscopy (SEM). Each new species is compared with morphologically similar taxa, and an updated identification key to the males of all Turkish representatives of the subgenus is also presented. These results contribute to a more comprehensive understanding of sarcophagid diversity across the Anatolian Peninsula and highlight the value of integrative morphological approaches in revealing previously undescribed species richness within this taxonomically challenging group.

## 2. Materials and Methods

### 2.1. Specimen Collection and Depository

Material examined in this study has been deposited in the collection of the Plant Protection Department Yozgat Bozok University, Yozgat, Türkiye (PPDY) and the Natural History Museum Denmark of Copenhagen University, Denmark (NHM-DK).

Specimens examined were collected from central and southern Anatolia in 1993, 1998, and 2016–2017 by sweep netting in open agricultural or semi-natural habitats. Collectors included Ş. Akman, G. Bakır, G. Pekbey, K. Kara, and D. Werner. Complete label data for each type specimen are provided verbatim in the species descriptions below.

### 2.2. Morphological Examination and Dissection

Before dissection, specimens were relaxed in a humidity chamber for 24–48 h to prevent damage to fragile structures. The terminalia of each specimen were carefully detached from the abdomen using fine forceps and insect pins under a Leica S8 APO stereomicroscope (Leica Microsystems, Wetzlar, Germany).The dissected terminalia of the holotype were macerated in 10% aqueous potassium hydroxide (KOH) solution prepared from potassium hydroxide pellets (EMPLURA®, Merck KGaA, Darmstadt, Germany) for 12 h to remove soft tissue, rinsed thoroughly with laboratory-prepared distilled water, and transferred to glycerol 99.96% FCC, FG (vegetable glycerol; Sigma-Aldrich, product no. W252506, St. Louis, MO, USA) for temporary mounting and detailed examination. Morphological observations and measurements were performed under a Leica S8 APO stereomicroscope (Leica Microsystems, Wetzlar, Germany). Photographs were taken using a Leica DFC450 digital camera mounted on a Leica M125 stereomicroscope (Leica Microsystems, Wetzlar, Germany) and processed through image stacking in Helicon Focus Pro version 8.3.11 (Helicon Soft Ltd., Kharkiv, Ukraine) to achieve extended depth of field.

### 2.3. Scanning Electron Microscopy (SEM)

For ultrastructural examination, the air-dried terminalia of the holotype were mounted on aluminium stubs using carbon double-sided adhesive tape and sputter-coated with gold to enhance conductivity and image contrast. Specimens were examined and imaged using a FEI Quanta 450 FEG scanning electron microscope (FEI Company, Hillsboro, OR, USA) operated under high vacuum conditions at the Science and Technology Application and Research Centre of Yozgat Bozok University (BILTEM), Yozgat, Türkiye.

After SEM examination, the terminalia were removed from the aluminium stubs, rinsed, transferred to glycerol vials, and pinned with the respective holotype specimens for permanent curation.

### 2.4. Illustrations

Line drawings of diagnostic structures of the species were prepared by the late Ruth M. Blackith with the use of a camera lucida and provided to the authors as unpublished original artwork.

### 2.5. Terminology and Taxonomic Framework

Terminology follows [4,6,14]. All new taxa described herein have been registered in the Official Registry of Zoological Nomenclature (ZooBank) and assigned unique Life Science Identifiers (LSIDs) in accordance with the requirements of the International Code of Zoological Nomenclature (ICZN) for electronic publications [16].

Label data for type specimens is transcribed verbatim. Within a single label, lines are separated by commas, individual labels are delimited by a double forward slash (//), and editorial remarks are enclosed in square brackets ([ ]).

## 3. Results

### 3.1. Sarcophaga (Sarcophaga) hayati sp. nov.

**Figure 1:**
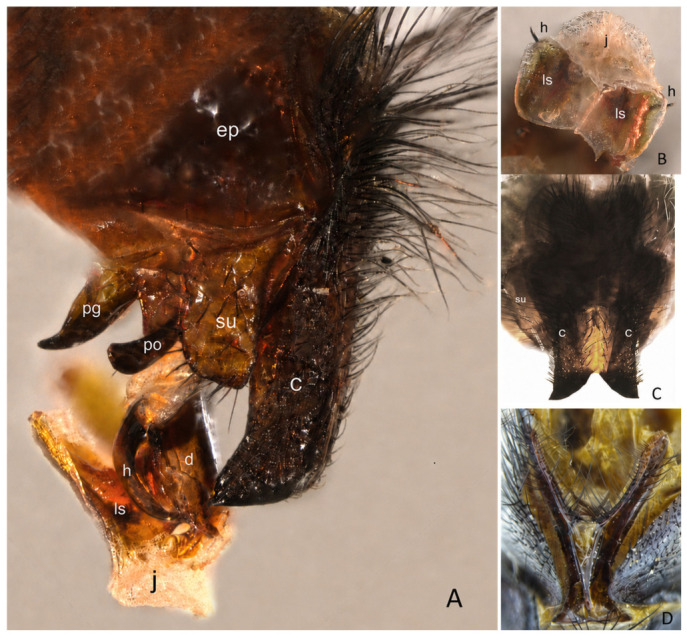
*Sarcophaga* (*Sarcophaga*) *hayati* Pekbey & Pape, sp. nov., male, holotype, [Antalya, Serik]. (**A**) Terminalia, left lateral view. (**B**) Distiphallus, ventral view. (**C**) Cerci and surstyli, dorsal view. (**D**) ST5. Abbreviations: c, cerci; d, distiphallus; ep, epandrium; h, harpes; j, juxta; ls, lateral stylus; pg, pregonite; po, postgonite; su, surstylus.

**Figure 2:**
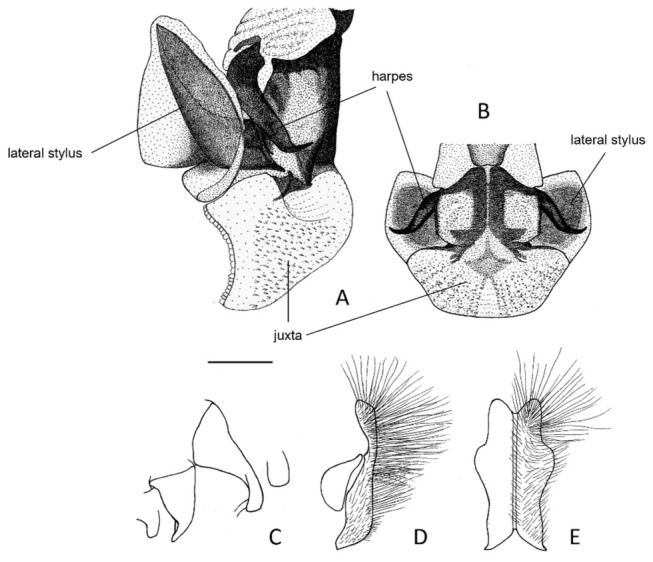
*Sarcophaga* (*Sarcophaga*) *hayati* Pekbey & Pape, sp. nov., male paratype [Muğla, Marmaris]. (**A**) Distiphallus, left lateral view. (**B**) Distiphallus dorsal view. (**C**) Pre- and postgonites, lateral view. (**D**) Cercus and surstylus, left lateral view. (**E**) Cerci, dorsal view. Scale bar: (**A**,**B**) 0.50 mm.

**Figure 3:**
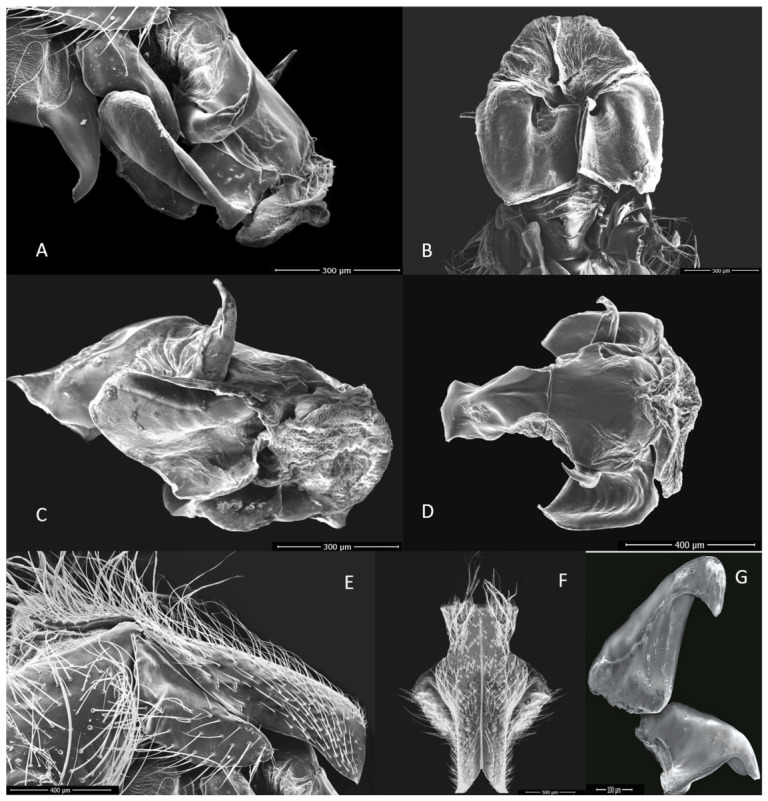
*Sarcophaga* (*Sarcophaga*) *hayati* Pekbey & Pape, sp. nov., male paratype [Mersin, Mut]. (**A**) Distiphallus, right lateral view. (**B**) Distiphallus, ventral view. (**C**) Distiphallus, right lateroventral view. (**D**) Distiphallus, ventral view. (**E**) Cercus and surstylus, lateral view. (**F**) Cerci and surstyli, dorsal view. (**G**) Pre- and postgonites, lateral view.

ZooBank LSID: urn:lsid:zoobank.org:pub:29871BCE-D936-4C9C-90C7-631528708A40

(Figure 1, Figure 2 and Figure 3)

**Type material. Holotype:** ♂, TR//Antalya province [southern Anatolia], Serik district [sweep net], 311 m, 37°11′42″ N 30°58′42″ E, 22.vi.2017, leg. Ş. Akman [printed on white paper]//Holotype ♂ *Sarcophaga* (*Sarcophaga*) *hayati* sp. nov., Det. Pekbey & Pape, 2026 [printed on red paper], [PPDY]. Paratype: ♂, TR//Mersin province [southern Anatolia], Mut district [sweep net], 351 m, 36°54′40″ N 33°43′57″ E, 05.vi.2016, leg. G. Bakır [printed on white paper] [PPDY]. *Sarcophaga* (*Sarcophaga*) *hayati* sp. nov., Det. Pekbey & Pape, 2026 [printed on red paper]. Paratype: ♂, TR//Marmaris [Muğla], 20.ix.1993, D. Werner [NHM-DK].

**Description.** Male. Length 11.7 mm.

Body colour. Head black with dense golden pollinosity. Eyes bare, red. Antennae black, postpedicel black with grey pruinescence, arista dark brown. Frontal vitta and orbital triangle dark grey. Fronto-orbital and parafacials black with golden pollinosity. Facial ridge brown and bare. Genal groove brown, genal dilation golden pollinose. Prementum black. Palpi black with brown tip. Thorax black, grey pollinosity, scutum with three black longitudinal stripes. Legs black. Wings hyaline, tegula black, basicosta yellow, calypters whitish. Abdomen black with silvery chequered pattern pollinosity. Protandrial segment black with grey pollinosity, epandrium, cerci and gonites dark brown, surstyli light brown.

**Head.** Frons at narrowest point 0.72 times as wide as an eye in dorsal view. Arista about 0.62 times as long as postpedicel, plumose on basal 3/5. Pedicel 0.43 times as long as postpedicel. Frontal vitta 0.50 times as wide as frons, widened at vertex. Frontal bristles 10 pairs. Fronto-orbital plate with a row of weak bristles close to eye margin. Parafacial plate 0.46 times as wide as minimum lateral eye width with scattered short setulae in lower half. Inner vertical setae long and stout, almost 2.0 times as long as outer vertical setae. Outer vertical setae slightly stronger than post ocular setae. Gena 0.32 times as high as in lateral minimum an eye height with a few scattered and fine apical bristles. Two rows of black occipital bristles behind post-ocellar bristles, post-occipital and postgenal hairs long, pale. Lower facial margin slightly visible below vibrissae with a few scattered apical hairs. Prementum long, 2.8 times as long as wide.

**Thorax**. Acrostichals 0 + 1, dorsocentrals 4 + 4, intra-alars 2 + 2, supra-alars 1 + 2, postalars 2, postpronotals 2 + 1, discal scutellar setae 1 pair, apical scutellar setae 1 pair, lateral scutellar setae 2 pairs on each side. Anepisternum setulose with a row of 5 strong setae. Katepisternum with 3 strong setae and a group of fine setulae.

**Wings**. Costal spine absent. R1 bare, R4 + 5 with a row of short setulae at base, cell r4 + 5 open at wing margin. Second costal sector 1.4 times as long as 4th costal sector.

**Legs**. Fore tibia with 3 posterodorsal setae. Mid femur with 5 strong posterodorsal setae and a row of short, stout ventral comb-like setae. Mid tibia with 2 posterodorsal and 1 anterodorsal setae (right mid tibia broken). Hind trochanter with fine and long anterior setulae. Hind femur with two rows of anterodorsal and 1 row of posteroventral setae. Hind tibia with 2 posterodorsal, 2 posteroventral and 1 anterodorsal setae.

**Terminalia.** Sternite 5 deeply indented, Y-shaped, median part protruding in profile, lateral process narrow and elongate and connected with a median triangular window (Figure 1D). Protandrial segment with short marginal bristles. Epandrium subquadrate to trapezoidal in lateral view (Figure 1A). Cerci short and stout curved at a wide angle, with a short swelling and setulose posterior surface, ventral outline shallowly S-shaped and blunt-ended in lateral view (Figure 1C, Figure 2D,E and Figure 3F). Surstyli large, subtriangular with a few weak hairs on posterior margin (Figure 1C and Figure 2D). Gonites nearly equal in length, pregonite conical, widened basally and tapering apically with blunt tip. Postgonite broad, curved at right angles and terminates in an acute peak with two fine setae (Figure 2C). Basiphallus short and oblique, nearly concealed by cerci. Distiphallus broadly connected to basiphallus in lateral view. Paraphallus and harpes well sclerotized; harpes horn-shaped and extending distal to the dorsal margin of distiphallus. Lateral styli with greatly enlarged membranous edge. Juxta membranous and strongly expanded anteriorly together with membranous edge of lateral styli. Vesica and ventral plate undeveloped (Figure 1A, Figure 2A,B, and Figure 3A–D).

**Etymology.** The specific epithet *hayati* is a patronym honouring Prof. Dr. Rüstem Hayat (Isparta University of Applied Sciences, Türkiye), in recognition of his contributions to Turkish Diptera and for his guidance and support in the academic career of the first author.

**Female.** Unknown.

**Distribution.** Palaearctic. Türkiye (Anatolia).

**Biology.** Larval biology unknown.

**Habitat:** Specimens were collected in open agricultural and semi-natural habitats: the holotype from Serik, Antalya (311 m a.s.l.) and paratypes from Mut, Mersin (351 m a.s.l.) and Marmaris, Muğla, all in southern Türkiye.

**Differential diagnosis**. *Sarcophaga* (*Sarcophaga*) *hayati* sp. nov. is most similar to *Sarcophaga* (*S*.) *pagensis* Baranov, 1939 and *S*. (*S*.) *trabzonensis* Pekbey et al., 2011 [17]. The species can be distinguished from these primarily by the morphology of the vesica, harpes and juxta, as well as the configuration of the lateral styli and associated phallic structures.

In *S*. (*S*.) *hayati*, the harpes are well sclerotized, distinctly horn-shaped, and project independently above the dorsal margin of the distiphallus. The distiphallus is broadly connected to the basiphallus, forming a relatively compact phallic complex. The juxta is broadly membranous and fully expanded anteriorly, forming a continuous membranous complex with the enlarged membranous accessory lobes of the lateral styli. The lateral styli have greatly enlarged membranous expansions, and both the vesica and ventral plate are weakly developed or indistinct.

In contrast, *S*. (*S*.) *trabzonensis* has a strongly sclerotized vesica that is markedly elongate (approximately 2.2 times longer than wide), apically curved upward, and associated with a conspicuous ventral membrane projecting below the vesica, clearly visible in lateral view. The juxta is thin, membranous, and closely appressed to the lateral styli, covering most of their surface and leaving only the apical portion exposed; it also forms an anterodorsal projecting membranous structure with fine ridges and points. The lateral styli are strongly curved, partially toothed along the ventral margin, and their tips are expanded and directed ventrally and posteriorly, reaching approximately the midpoint of the vesica. The ventral plate is moderately developed and clearly defined, and the harpes, although largely concealed by the ventral membrane, are discernible apically (see [17]).

*Sarcophaga* (*S*.) *pagensis* differs from both species by having the vesica composed of a pair of flattened plates directed laterally, with widely separated tips, rather than a single horn-shaped or elongate structure. The ventral plate is not developed. The juxta is large and conspicuous, forming a thin membrane greatly expanded anteroventrally, but not, or only slightly, extending above the dorsal margin of the distiphallus. In contrast to *S*. (*S*.) *trabzonensis*, the juxta is thicker and projects anteriorly away from the lateral styli, rather than being closely appressed to them. The lateral styli in *S*. (*S*.) *pagensis* have greatly expanded tips that reach or extend beyond the level of the base of the vesica. Additionally, the harpes are undeveloped, and the cercus lacks a well-defined subapical ventral swelling (see [10]).

Taken together, the combination of (i) vesica and harpes shape and orientation, (ii) degree and position of juxtal development, and (iii) configuration and extent of the lateral styli provides reliable separation of *S*. (*S*.) *hayati*, from the morphologically similar *S*. (*S*.) *trabzonensis* and *S*. (*S*.) *pagensis*.

### 3.2. Sarcophaga (Sarcophaga) karai sp. nov.

**Figure 4:**
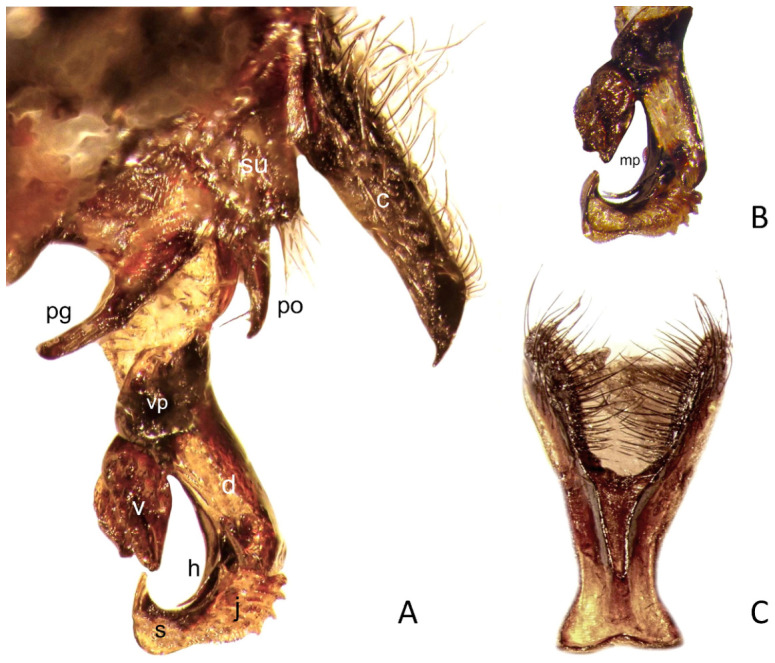
*Sarcophaga* (*Sarcophaga*) *karai* Pekbey & Pape, sp. nov., male, holotype [Yozgat. Çekerek]. (**A**) Terminalia, left lateral view. (**B**) Distiphallus, left lateral view. (**C**) ST5. Abbreviations: c, cerci; d, distiphallus; h, harpes; j, juxta; mp, median process; pg, pregonite; po, postgonite; s, stylus; su, surstylus; v, vesica; vp, ventral plate.

**Figure 5:**
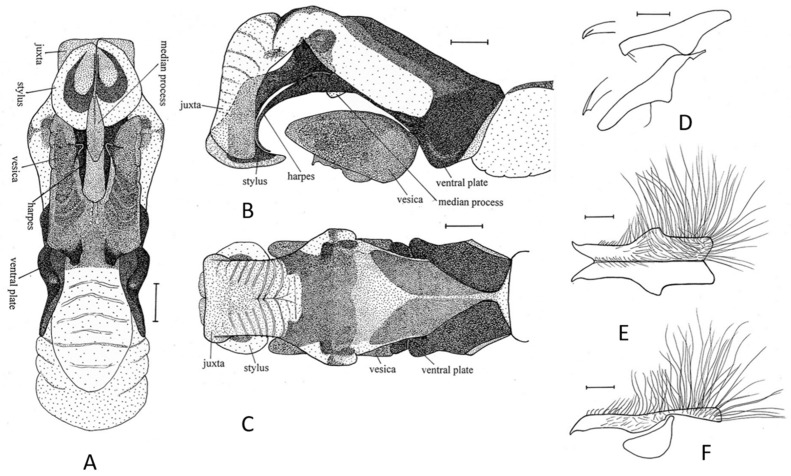
*Sarcophaga* (*Sarcophaga*) *karai* Pekbey & Pape, sp. nov., male paratype [Tokat, Turhal]. (**A**) Distiphallus, ventral view. (**B**) Distiphallus, right lateral view. (**C**) Distiphallus, dorsal view. (**D**) Pre- and postgonites, right lateral view. (**E**) Cerci, dorsal view. (**F**) Cercus and surstylus, right lateral view. Scale bars: (**A**–**C**) 0.50 mm; (**D**–**F**) 0.10 mm.

**Figure 6:**
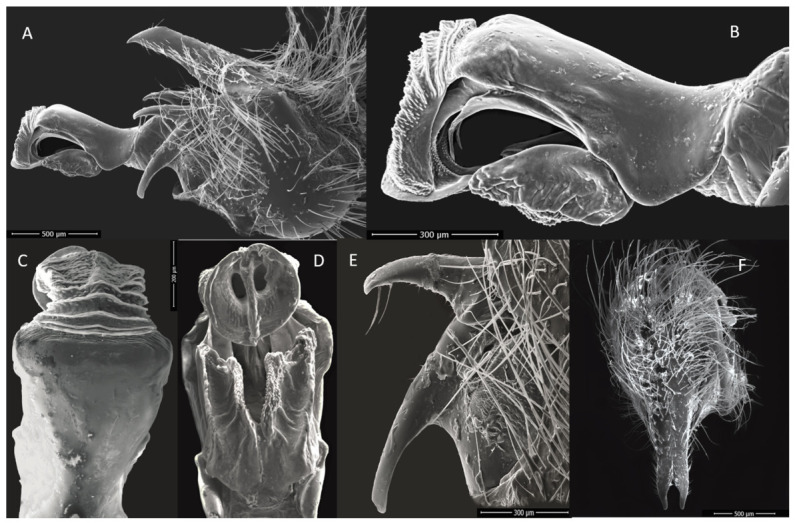
*Sarcophaga* (*Sarcophaga*) *karai* Pekbey & Pape, sp. nov., male paratype [Yozgat, Çekerek]. (**A**) Terminalia, right lateral view. (**B**) Distiphallus, right lateral view. (**C**) Distiphallus, dorsal view. (**D**) Distiphallus, ventral view. (**E**) Pre- and postgonites, right lateral view. (**F**) Cerci, dorsal view.

ZooBank LSID: urn:lsid:zoobank.org:pub:A597A2F2-4313-4173-8D13-AE466D3EDDB2

(Figure 4, Figure 5 and Figure 6)

**Type material. Holotype:** ♂, TR//Yozgat province [Central Anatolia], Çekerek district [sweep net], 994 m, 40°04′19″ N 35°30′52″ E, 18.vii.2016, leg. G. Pekbey [printed on white paper]//Holotype ♂ *Sarcophaga* (*Sarcophaga*) *karai* sp. nov., Det. Pekbey & Pape, 2026 [printed on red paper], [PPDY]. Paratype: ♂, TR//Tokat-Turhal, 19/6 [19.vi.] 1998, K. Kara [leg.] [NHM-DK].

**Description**. Male. Length 10.8 mm.

**Body colour.** Head black with silver pollinosity. Eyes bare, red. Antennae, arista and postpedicel black. Postpedicel with brown pruinescence. Frontal vitta and orbital triangle dark brown, blackish anteriorly above lunula. Fronto-orbital and parafacials black with silver pollinosity. Facial ridge brown and bare. Genal groove and genal dilation black silvery pollinose. Prementum black. Palpi black with brown tip. Thorax black, grey pollinosity, scutum with three black longitudinal stripes. Legs black. Wings hyaline, tegula black, basicosta yellow, calypters yellowish. Abdomen black with silvery chequered pattern pollinosity. Protandrial segment black with pale grey pollinosity, epandrium, cerci, gonites and surstyli black.

**Head.** Frons at narrowest point 0.78 times as wide as an eye in dorsal view. Arista about 0.66 times as long as postpedicel, plumose on basal 2/5. Pedicel 0.51 times as long as postpedicel. Frontal vitta 0.46 times as wide as frons. Frontal bristles 8 pairs. Fronto-orbital plate with a row of hairy bristles close to eye margin. Parafacial plate 0.40 times as wide as minimum lateral eye width with a row of 4 setae in lower half of eye margin. Inner vertical setae long (1/3 broken), outer vertical setae broken. Gena 0.22 times as long as minimum an eye height in lateral; setulae on gena and post gena black. Two rows of black occipital bristles behind post-ocellar bristles, post-occipital and postgenal hairs long and pale. Lower facial margin hardly visible, vibrissae broken. Prementum long, 3.8 times as long as wide.

**Thorax.** Acrostichals 0 + 1, dorsocentrals 2 + 3, intra-alars 1 + 2, supra-alars 2 + 3, postalars 2, postpronotals 2 + 1, discal scutellar 1 pair, scutellar apicals 1 pair, scutellar laterals 2 pairs on each side. Anepisternum setulose with a row of 4 strong setae. Katepisternum with 3 strong and a bunch of fine setulae.

**Wings.** Costal spine absent. R1 bare, R4 + 5 with a row of 4 short setulae at base, cell r4 + 5 open at wing margin. 2nd costal sector 1.7 times as long as 4th costal sector.

**Legs.** Fore tibia with 1 posteroventral seta. Mid femur with a row of short and stout ventral comb-like setae. Mid tibia with 1 posteroventral and 1 anteroventral seta. (Left mid leg broken). Hind trochanter with fine and long anterior setulae. Hind femur with two rows of anterodorsal and 1 row of posteroventral setae. Hind tibia with 3 posterodorsal, 2 posteroventral and 1 anterodorsal setae.

**Abdomen.** Tergite 1 + 2 and tergite 3 bare. Tergite 4 with a pair of median marginal bristles. Tergite 5 with a row of strong marginal setae. Sternite 1–4 exposed.

**Terminalia.** Sternite 5 deeply indented, Y-shaped, median part protruding in profile, lateral process narrow and elongate and connected with a wide median membrane (Figure 4C). Protandrial segment with long and stout marginal bristles. Epandrium short, subquadrate in lateral view (Figure 4A and Figure 6A). Cerci narrow and stout in lateral view with numerous long and hair-like setae on posterior surface; tip of cerci projecting beyond ventral margin with a small subapical ventral swelling and narrow hook (Figure 4A, Figure 5E,F and Figure 6A,F). Surstyli subtriangular, expanding anteriorly with a few weak hairs on posterior margin (Figure 4A). Pregonite long and narrow with blunt tip. Postgonite short and straight, slightly inclined tip with two subapical setae (Figure 5D and Figure 6E). Basiphallus short and stout. Ventral plates well developed. Styli large and distinctly curving above vesica, not projecting beyond margin of ventral plates in lateral view. Styli and juxta forming an angle of 85–95° with longitudinal axis of distiphallus. Juxta membranous and in lateral view slightly notched at the point of curvature. Vesica bifid, projecting beyond styli and ventral plates; narrower in lateral view (about 1.5× as long as wide). Harpes thin and long, slightly slanting beneath styli (Figure 4A,B, Figure 5A,B and Figure 6B–D).

**Etymology.** The specific epithet *karai* is a patronym honouring Prof. Dr. Kenan Kara (Tokat Gaziosmanpaşa University, Türkiye) in recognition of his contributions to Turkish Diptera, and for collecting the paratype.

**Female.** Unknown.

**Distribution.** Palaearctic. Türkiye (Anatolia).

**Biology.** Larval biology unknown.

**Habitat.** Specimens were collected in open agricultural and semi-natural habitats: the holotype from Çekerek, Yozgat (Central Anatolia, 994 m a.s.l.) and the paratype from Tokat-Turhal, both in north-central Türkiye.

**Differential diagnosis.** *Sarcophaga* (*Sarcophaga*) *karai* sp. nov. is closest to *S*. (*S*.) *variegata* (Scopoli, 1763) and *S*. (*S*.) *croatica* Baranov, 1941 in having a distiphallus with well-developed ventral plates. *S.* (*S.*) *karai* sp. nov. differs from *S.* (*S.*) *variegata* by the styli being distinctly curved dorsally above the vesica and not extending beyond the ventral plates in lateral view, whereas in *S*. (*S*.) *variegata* the styli are narrower, situated approximately at a right angle to the longitudinal axis of the distiphallus, and project beyond the ventral plates.

The juxta of *S.* (*S.*) *karai* is membranous and slightly notched at the point of curvature, whereas in *S.* (*S.*) *variegata* it bears a distinct angular projection. The vesica of *S.* (*S*.) *karai* is bifid and projects beyond the styli and ventral plates, being relatively broad in lateral view (about 1.5× as long as wide), while in *S.* (*S.*) *variegata* the vesica is narrow, bean-shaped, distinctly longer than broad (about 1.7× as long as wide), and does not reach the apex of the styli. In addition, the cerci of *S.* (*S.*) *karai* possess a narrower apical hook and a more pronounced subapical ventral swelling than those of *S.* (*S.*) *variegata* (see [10]). *S.* (*S*.) *karai* sp. nov. can be separated from *S*. (*S*.) *croatica* by the shape of the styli, juxta and vesica. In *S*. (*S*.) *karai*, the styli are curved above the vesica and form an angle of 85–95° with the longitudinal axis of the distiphallus, whereas in *S*. (*S*.) *croatica* the styli are directed more laterally, forming an angle of 135–140°.

The juxta in S. (S.) karai is weakly notched at the point of curvature, while in *S.* (*S.*) *croatica* it has a smooth outline lacking any projection or serration. The vesica of *S.* (*S.*) *karai* is bifid and relatively narrow in lateral view (about 1.5× as long as wide), whereas that of *S.* (*S.*) *croatica* is broader (about 1.4× as long as wide) and not distinctly bifid. Furthermore, the distiphallus of *S.* (*S.*) *karai* is more elongate and less thickset than in *S.* (*S.*) *croatica*, and the cercal hook is broader and less strongly associated with a pronounced subapical ventral swelling (see [10]).

Taken together, the combination of (i) the angle formed by the lateral styli with the longitudinal axis of the distiphallus, (ii) the shape, proportions, and degree of bifurcation of the vesica, and (iii) the outline and development of the juxta at the point of curvature provides reliable separation of *S.* (*S.*) *karai* sp. nov. from the morphologically similar *S.* (*S.*) *variegata* and *S.* (*S.*) *croatica*. Additional support for the distinctness of *S.* (*S.*) *karai* is provided by the configuration of the cercal apex, particularly the narrower apical hook and more pronounced subapical ventral swelling, which further distinguish it from both allied species.

## 4. Discussion

Until now, the subgenus *Sarcophaga* Meigen, 1826 was represented in Türkiye by six known species: *S*. (*S*.) *bergi* Rohdendorf, 1937; *S*. (*S*.) *carnaria* (Linnaeus, 1758); *S*. (*S*.) *croatica* Baranov, 1941; *S*. (*S*.) *lehmanni* Müller, 1922; *S*. (*S*.) *trabzonensis* Pekbey, Hayat, Richet & Blackith, 2011; and *S*. (*S*.) *variegata* (Scopoli, 1763) [17,18,19,20]. The description of *S*. (*S*.) *karai* sp. nov. and *S*. (*S*.) *hayati* sp. nov. brings the total number of subgenus representatives recorded from Türkiye to eight.

The two new species described here differ markedly in the configuration of their phallic complexes. *Sarcophaga* (*S*.) *karai* sp. nov. is most closely related to *S*. (*S*.) *variegata* and *S*. (*S*.) *croatica* due to its well-developed ventral plates. It can be reliably separated from both by a combination of characters in the styli, juxta, and vesica. *Sarcophaga* (*S*.) *hayati* sp. nov. belongs to a morphologically distinct group characterized by greatly enlarged membranous accessory lobes of the lateral styli, a horn-shaped vesica projecting above the dorsal margin of the distiphallus, and a broadly expanded membranous juxta. These characters are shared with *S*. (*S*.) *trabzonensis* and *S*. (*S*.) *pagensis*, from which *S.* (*S.*) *hayati* sp. nov. can be distinguished by the features detailed in the differential diagnosis above. Notably, *S*. (*S*.) *trabzonensis*, described previously from northeastern Türkiye [17], is also a Palaearctic endemic known exclusively from Türkiye, and the addition of *S*. (*S*.) *hayati* sp. nov. means that Türkiye has now contributed three endemic species of the subgenus, suggesting that the Anatolian Peninsula harbours high diversity within this group.

The present results further confirm the central role of male genital morphology, particularly the structure of the distiphallus, in delimiting species within *Sarcophaga* (*Sarcophaga*). Characters such as the angle formed by the lateral styli with the longitudinal axis of the distiphallus, the shape and proportions of the vesica, the outline and degree of development of the juxta, and the configuration of the cercal apex collectively provide a consistent and reliable basis for species identification. The complementary use of scanning electron microscopy in this study was particularly valuable for resolving fine ultrastructural details, including surface textures and three-dimensional configurations of the distiphallic sclerites that cannot be adequately characterized under stereomicroscopy alone. This confirms the utility of SEM as a standard tool in integrative morphological studies of the family.

To facilitate identification of all currently known Turkish representatives of the subgenus, an updated identification key to the males of all eight species is provided in Appendix A. The key is based primarily on characters of the distiphallus and is intended as a practical reference for future faunistic, forensic, and ecological studies in the region, although the reliability of some characters (e.g., vesica proportions) may be reduced in dried specimens, as previously noted [15]. Future work combining morphological revision, molecular approaches, and expanded geographic sampling will be essential for a more complete understanding of sarcophagid diversity and biogeographic patterns across the Anatolian Peninsula and the broader western Palaearctic.

The Anatolian Peninsula, located at the biogeographical crossroads of Europe, Asia, and the Middle East, encompasses an exceptional range of climatic zones, altitudinal gradients, and ecological systems [21]. The discovery of two new species of *Sarcophaga* (*Sarcophaga*) Meigen, 1826, from material collected in central and southern Anatolia, along with the earlier description of *S*. (*S*.) *trabzonensis* from the northeastern part of the country, indicates that the Turkish fauna of this subgenus may be substantially more diverse than current records.

## 5. Conclusions

The present study describes two new species of *Sarcophaga* (*Sarcophaga*) from Türkiye, providing morphological diagnoses, illustrations, scanning electron microscopy (SEM) documentation of the male terminalia, and a revised identification key to the Turkish males of the subgenus. The addition of these taxa brings the total number of recorded representatives of the subgenus from Türkiye to eight, contributing to a growing body of evidence for the considerable sarcophagid diversity of the Anatolian Peninsula within the western Palaearctic. The revised key and diagnostic characters presented here are intended to facilitate future faunistic, forensic, and ecological research in the region.

## Data Availability

The original contributions presented in this study are included in the article. For more information, please contact the authors.

## Abbreviations

The following abbreviations are used in the figures and manuscript:

a.s.l.	above sea level
BILTEM	Science and Technology Application and Research Centre of Yozgat Bozok University, Yozgat, Türkiye.
c	cerci
d	distiphallus
ep	epandrium
h	harpes
j	juxta
ls	lateral stylus
mp	median process
NHM-DK	Natural History Museum Denmark of Copenhagen University, Denmark
pg	pregonite
po	postgonite
PPDY	Plant Protection Department, Yozgat, Bozok University, Yozgat, Türkiye
s	stylus
SEM	Scanning Electron Microscopy
ST5	abdominal sternite 5
su	surstylus
v	vesica
vp	ventral plate

## Appendix A. Key to Males of Sarcophaga (Sarcophaga) Meigen, 1826 Species from Türkiye

1. Vesica elongate, in lateral view more than twice as long as wide, with a distinct ventral membrane projecting below; juxta thin and flattened against lateral styli; lateral styli reaching approximately to midpoint of vesica (see Figure 1E in [17])................................. *Sarcophaga (S.) trabzonensis* Pekbey, Hayat, Richet & Blackith
− Vesica shorter, weakly developed, or differently configured, without a distinct ventral membrane projecting below; juxta broadly membranous or otherwise not thin and flattened against lateral styli; lateral styli not reaching approximately to midpoint of vesica......................................................................................................................................2
2. Harpes horn-shaped, directed dorsally and extending toward dorsal margin of distiphallus; vesica undeveloped or indistinct; juxta broadly expanded anteriorly; lateral styli with large membranous accessory lobes (Figure 1, Figure 2 and Figure 3)....... *Sarcophaga (S.) hayati* Pekbey & Pape, sp. nov.
− Harpes not horn-shaped and not directed toward dorsal margin of distiphallus; vesica developed; juxta not broadly expanded anteriorly; lateral styli without large membranous accessory lobes................................................................................... 3
3. Lateral styli forming an angle of 140–160° with longitudinal axis of distiphallus and rounded apically; vesica elongate, in lateral view more than 1.75× maximum width of ventral plate; distiphallus elongate (see Plate 076 D in [10])................... *Sarcophaga (S.) carnaria* (Linnaeus)
− Lateral styli forming an angle of less than 140° with longitudinal axis of distiphallus and not rounded apically in the above manner; vesica shorter relative to ventral plate width; distiphallus not elongate ......................................................................................... 4
4. Vesica markedly broad (approximately 1.2–1.3× as long as wide), strongly rounded and entire; distiphallus short, robust and thickset; lateral styli strongly curved and robust; juxta with broad, smoothly rounded lobes, without serration or notch (see Figure 7 in [22])....................................................... *Sarcophaga (S.) bergi* Rohdendorf
− Vesica narrower (approximately 1.4× or more as long as wide), bifid or not strongly rounded and entire; distiphallus more elongate or less thickset; lateral styli less robust or configured differently; juxta without broad, smoothly rounded lobes in the above combination ............................................................................................................................... 5
5. Lateral styli oriented at approximately a right angle (85–95°) to longitudinal axis of distiphallus and projecting beyond ventral plates; juxta with a distinct angular projection at point of curvature; vesica narrow, bean-shaped, distinctly longer than wide (approximately 1.7×), not attaining apex of lateral styli (see Plate 085 D, E in [10])............................................................................... *Sarcophaga (S.) variegata* (Scopoli)
− Lateral styli forming an angle greater than 95° with longitudinal axis of distiphallus or not projecting beyond ventral plates; juxta without a distinct angular projection at point of curvature; vesica broader, attaining or exceeding level of lateral styli ............................................................................................................................. 6
6. Lateral styli forming an angle of 95–110° with longitudinal axis of distiphallus; vesica subtriangulate; distiphallus in profile with a narrow membranal window centrally (see Plate 079 D, E in [10])............................................... *Sarcophaga (S.) lehmanni* Müller
− Lateral styli forming an angle greater than 110° with longitudinal axis of distiphallus or distinctly curved dorsally above vesica; vesica not subtriangulate; distiphallus in profile without a narrow membranal window centrally .................................................. 7
7. Lateral styli forming an angle of 135–140° with longitudinal axis of distiphallus; juxta with smooth outline, without serration or projection; vesica relatively broad (approximately 1.4× as long as wide), not bifid; distiphallus thickset (see Plate 077 D, E in [10])............................................................................... *Sarcophaga (S.) croatica* Baranov
− Lateral styli distinctly curved dorsally above vesica, forming an angle of 85–95° with longitudinal axis of distiphallus, not projecting beyond margin of ventral plates in lateral view; juxta membranous, slightly notched at point of curvature; vesica bifid, projecting beyond lateral styli and ventral plates, moderately narrow in lateral view (approximately 1.5× as long as wide); distiphallus more elongate, less thickset (Figure 4, Figure 5 and Figure 6) ................................................. *Sarcophaga (S.) karai* Pekbey & Pape, sp. nov.
